# Dabcyl as a Naked Eye Colorimetric Chemosensor for Palladium Detection in Aqueous Medium

**DOI:** 10.3390/molecules28166111

**Published:** 2023-08-17

**Authors:** Cátia D. F. Martins, M. Manuela M. Raposo, Susana P. G. Costa

**Affiliations:** Centre of Chemistry, University of Minho, Campus de Gualtar, 4710-057 Braga, Portugal; catiadf_martins@hotmail.com (C.D.F.M.); mfox@quimica.uminho.pt (M.M.M.R.)

**Keywords:** azo dye, colorimetric chemosensor, Dabcyl, naked eye, iron, palladium, tin

## Abstract

Industrial activity has raised significant concerns regarding the widespread pollution caused by metal ions, contaminating ecosystems and causing adverse effects on human health. Therefore, the development of sensors for selective and sensitive detection of these analytes is extremely important. In this regard, an azo dye, Dabcyl **2**, was synthesised and investigated for sensing metal ions with environmental and industrial relevance. The cation binding character of **2** was evaluated by colour changes as seen by the naked eye, UV-Vis and ^1^H NMR titrations in aqueous mixtures of SDS (0.02 M, pH 6) solution with acetonitrile (99:1, *v*/*v*). Out of the several cations tested, chemosensor **2** had a selective response for Pd^2+^, Sn^2+^ and Fe^3+^, showing a remarkable colour change visible to the naked eye and large bathochromic shifts in the UV-Vis spectrum of **2**. This compound was very sensitive for Pd^2+^, Sn^2+^ and Fe^3+^, with a detection limit as low as 5.4 × 10^−8^ M, 1.3 × 10^−7^ M and 5.2 × 10^−8^ M, respectively. Moreover, comparative studies revealed that chemosensor **2** had high selectivity towards Pd^2+^ even in the presence of other metal ions in SDS aqueous mixtures.

## 1. Introduction

The extensive use of metal ions in many industries has led to their accumulation in the environment, thus emerging as a considerable source of pollution and a significant threat to human health and other living organisms. Palladium, for instance, is widely applied in various materials, including electric and electronic equipment, fuel cells, dental appliances, jewellery and catalysts [1]. The highest demand for palladium has been in the automotive sector, due to its use in vehicle exhaust catalysts (VECs) to convert noxious gases emitted from automobile exhaust into less harmful substances. However, the extensive use of this metal in VECs has resulted in the release of palladium particles into the environment. Consequently, the dispersion of these particles through road dust has increased the palladium concentration in various environmental matrices, including soil, water sources, air and plants [2,3,4,5].

On the other hand, palladium-catalysed reactions are indispensable in the pharmaceutical industry, particularly in the preparation of complex drugs. Residual palladium may be found in final products, presenting a potential health hazard [6]. It has been reported that palladium is capable of binding to thiol-containing amino acids, proteins, DNA and other macromolecules and thereby may seriously disturb numerous cellular processes [1,2]. Very recent reports on new sensors for palladium confirm the interest in its detection [7,8,9].

Tin is also widely employed in a variety of industries. Sn(II) as a fluoride, chloride, or citrate complex is commonly found in different products, such as toothpaste, mouthwash, food and beverage cans, food additives and biocides. Additionality, SnCl_2_ is an extremely useful catalyst and reducing agent in the preparation of indole and coumarin derivatives. Although tin is an essential trace element for human beings, involved in human growth factors and cancer prevention, an excessive accumulation of Sn ions can result in adverse effects on the respiratory, digestive and nervous systems [10,11,12]. 

Among the transition metal ions, iron is the most abundant essential trace element in the human body, playing crucial roles in various cellular processes, including metabolism, electron transport, and DNA synthesis. Its deficiency or overload has a toxic effect on human health and is related to various disorders, including anaemia, hemochromatosis, and neurodegenerative diseases such as Alzheimer’s and Parkinson’s [13,14,15].

Despite the importance of the above-mentioned cations in biological processes and industrial fields, their wide use and improper disposal have resulted in various detrimental effects on ecosystems and human well-being. Hence, the development of effective and accurate methods for metal ion detection is of utmost importance.

Nowadays, colorimetric sensing methods are extremely attractive for biological and environmental applications due to their low cost, simplicity, high sensitivity and selectivity, as well as real-time response, without requiring advanced equipment. Colorimetric chemosensors allow qualitative detection of the target analyte by naked-eye-perceivable colour changes, while quantification can be performed using spectrophotometric analysis [16,17]. Their use provides a versatile and practical approach for diverse areas, including environmental monitoring, biomedical diagnostics, and food safety testing [18,19,20].

Among the colorimetric chemosensors, azo dyes play a crucial role due to their unique optical and structural properties. Their simple and cost-effective preparation methods, along with their ability to undergo noticeable colour changes, make them accessible and valuable probes for analyte sensing [21,22]. Additionally, the structural versatility of azo dyes allows the design and synthesis of chemosensors with tailored properties for specific analytes, enhancing their performance and applicability in various fields [17].

Dabcyl, a well-known azo dye (4-[[4′-(*N*,*N*-dimethylamino)phenyl]diazenyl]benzoic acid), has been employed in various biomolecular applications as a dark quencher in FRET-labelled probes [23,24,25]. This *para*-substituted azobenzene derivative exhibits a push–pull structure, which makes it highly promising for colorimetric sensing. So far, we have used Dabcyl in the synthesis of FRET-based probes to monitor protease activity [26], but herein we report the evaluation of Dabcyl **2** as a colorimetric chemosensor for industrially and environmentally important metal ions in aqueous medium. The interaction of this compound with various cations was studied by naked-eye-visible colour changes, UV-Vis absorption and ^1^H NMR spectroscopy in aqueous mixtures.

## 2. Results and Discussion

### 2.1. Synthesis

**Scheme 1:**
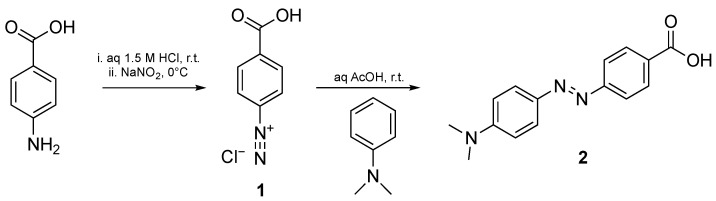
Synthetic route to Dabcyl **2**.

Dye **2** was prepared by an azo coupling reaction between the diazonium salt of 4-aminobenzoic acid **1** and *N*,*N*-dimethylaniline, as previously published by us (Scheme 1) [27]. This compound was obtained by precipitation as a dark red solid in high yield (92%), without further purification. The structure of Dabcyl **2** was confirmed by ^1^H and ^13^C NMR spectroscopy (Figures S1 and S2), which was in agreement with the previously reported assignment [27].

### 2.2. Preliminary Chemosensing Tests 

The need for detection and control of metal ions in environmental and industrial samples has increased in recent years; thus, the development of water-soluble probes is highly desired. Although Dabcyl **2** was poorly soluble in pure water, it was completely solubilised in the presence of the anionic surfactant sodium dodecyl sulphate (SDS). According to the literature, in aqueous media containing SDS, optical chemosensors and target ions can be organised inside the inner hydrophobic core of the SDS micelles, thereby ensuring chemosensor solubilisation as well as improving the sensing properties [28,29]. Therefore, the chromogenic behaviour of Dabcyl **2** was evaluated in aqueous mixtures of SDS with acetonitrile, namely in SDS (0.02 M, pH 6)–acetonitrile (99:1, *v*/*v*) solution.

**Figure 1:**
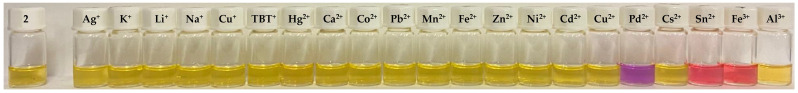
Colorimetric responses of **2** in SDS (0.02 M, pH 6)–acetonitrile 99:1 (*v*/*v*) solution (2 × 10^−5^ M) before and after the addition of 10 equiv. of various ions.

Firstly, the sensing ability of **2** towards various metal ions was qualitatively investigated by naked-eye-perceivable colour changes, through the addition of 10 equivalents of each cation to the solution of **2**. Among the 21 tested metal ions, chemosensor **2** responded selectively to Pd^2+^, Sn^2+^ and Fe^3+^, with a marked colour change from yellow to purple in the case of Pd^2+^ and from yellow to magenta after interaction with Sn^2+^ and Fe^3+^ (Figure 1). No obvious colorimetric response could be observed in the presence of other cations. Interestingly, the organotin cation (tributyltin, TBT) did not induce a colour change.

**Figure 2:**
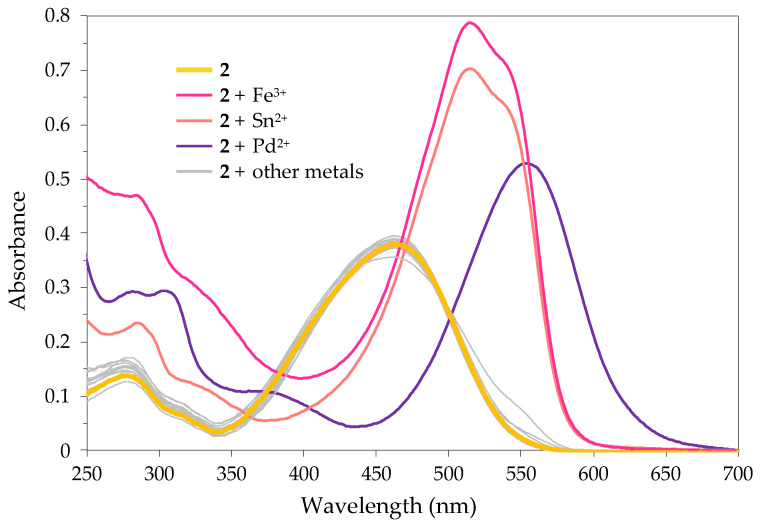
UV-Vis spectra of **2** in SDS (0.02 M, pH 6)–acetonitrile 99:1 (*v*/*v*) solution (2 × 10^−5^ M) before and after the addition of 10 equiv. of Ag^+^, K^+^, Li^+^, Na^+^, Cu^+^, TBT^+^, Hg^2+^, Ca^2+^, Co^2+^_,_ Pb^2+^, Mn^2+^, Fe^2+^, Zn^2+^, Ni^2+^, Cd^2+^, Cu^2+^, Pd^2+^_,_ Cs^2+^, Sn^2+^, Fe^3+^ and Al^3+^.

To further confirm the selectivity of **2**, UV-Vis absorption spectra were obtained before and after the addition of 10 equivalents of the selected cations (Figure 2). It was found that the ion-free solution exhibited a weak band at 278 nm (π-π*, *ε*_max_ = 6900 M^−1^ cm^−1^) and an intense absorption band at 462 nm (n-π*, *ε*_max_ = 19,000 M^−1^ cm^−1^). The latter is responsible for the yellow colour solution of Dabcyl **2** and could be ascribed to the intramolecular charge transfer (ICT) transition resulting from the push–pull effect between the electron-donating dimethylamino group and the carboxylic acid electron acceptor group. The absorption spectrum of **2** was altered upon interaction with Pd^2+^, Sn^2+^ and Fe^3+^, while other metal ions did not induce relevant changes. Notably, the addition of such ions resulted in large bathochromic shifts in the ICT band of **2**, from 462 nm to 555 nm (∆*λ* = 93 nm) in the case of Pd^2+^, and from 462 nm to 515 nm (∆*λ* = 53 nm) for Sn^2+^ and Fe^3+^. According to the large bathochromic shifts and concomitant naked-eye-visible colour changes, dye **2** could find application as a colorimetric chemosensor for Pd^2+^, Sn^2+^ and Fe^3+^ in aqueous mixtures. 

### 2.3. The Detection of Pd^2+^ in SDS Aqueous Solution 

#### 2.3.1. UV-Vis Titrations

**Figure 3:**
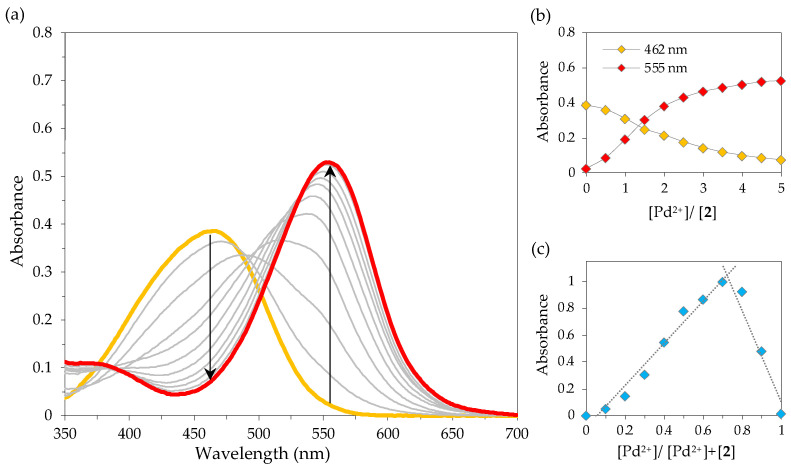
Absorption spectral changes of **2** with Pd^2+^ in SDS (0.02 M, pH 6)–acetonitrile 99:1 (*v*/*v*) solution (2 × 10^−5^ M): (**a**) UV-Vis titration of **2** upon gradual addition of Pd^2+^; (**b**) absorbance at 462 and 555 nm as a function of added Pd^2+^ equiv.; (**c**) Job’s plot for the complexation of **2** with Pd^2+^ at 555 nm.

Given the preliminary sensing results, the colorimetric response of chemosensor **2** in the presence of Pd^2+^ was evaluated in more detail by UV-Vis titrations in SDS (0.02 M, pH 6)–acetonitrile 99:1 (*v*/*v*) solution. Upon incremental addition of Pd^2+^, the absorption band at 462 nm decreased, while a new band appeared at 555 nm (Figure 3a, the colours of the curves relate to the colours of the solutions). The sensitivity of **2** towards Pd^2+^ was evident, since the addition of only five equivalents of cation was sufficient to achieve the maximal optical change (Figure 3b). The molar extinction coefficients were calculated at the wavelengths of maximum absorption for Dabcyl **2** (462 nm) and the complex Dabcyl–Pd (555 nm) and found to be *ε*_462_ = 19,300 M^−1^ cm^−1^ and *ε*_555_ = 1150 M^−1^ cm^−1^ (for **2**) and *ε*_462_ = 3700 M^−1^ cm^−1^ and *ε*_555_ = 26,450 M^−1^ cm^−1^ (for the complex).

In order to investigate the stoichiometry of complexation between chemosensor **2** and Pd^2+^, a Job’s plot analysis was performed in SDS aqueous solution. As can be seen in Figure 3c, a maximum absorbance was identified when the mole fraction of Pd^2+^ was ca. 0.7, indicating the formation of a complex with a ligand to metal 1:2 stoichiometry.

Based on the UV-Vis data, the detection limit (DL) of **2** for Pd^2+^ was determined using the equation DL = 3*σ*/*S*, where *σ* is the standard deviation of a blank solution and *S* is the slope between absorbance and Pd^2+^ concentration [30]. The DL for Pd^2+^ was found to be 5.4 × 10^−8^ M, which is lower than the WHO limit for palladium content in drugs (4.7 × 10^−5^ M (5 ppm) to 9.4 × 10^−5^ M (10 ppm)) [31]. The obtained DL is highly promising, as it demonstrates a close similarity to chemosensors reported in the literature for Pd^2+^ (Table 1). All the above results suggest that dye **2** could detect Pd^2+^ both qualitatively (naked eye) and quantitatively (UV-Vis spectroscopy) with high sensitivity in solutions containing large amounts of water.

#### 2.3.2. ^1^H NMR Studies

**Figure 4:**
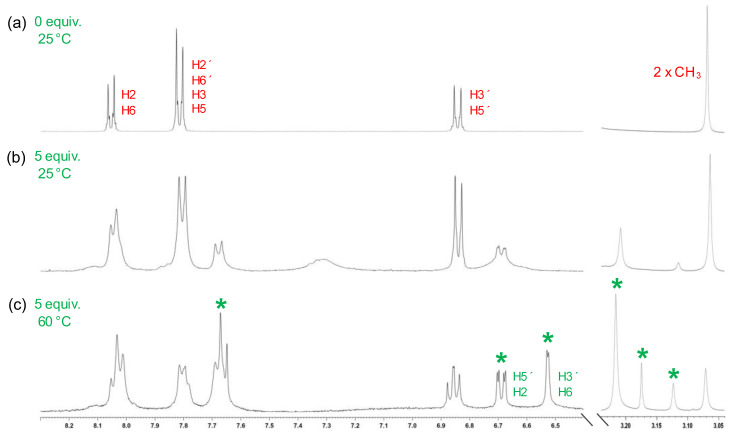
Partial ^1^H NMR spectra of **2** with the addition of Pd^2+^ (5 equiv.) in DMSO-*d_6_* at 25 and 60 °C (* indicates the new signals of the formed complex).

The binding mode between chemosensor **2** and Pd^2+^ was further investigated by recording the ^1^H NMR spectra before and after the sequential addition of palladium. Due to the low solubility of Dabcyl **2** in pure D_2_O, this study was performed in DMSO-*d_6_* solution. In the absence of Pd^2+^, the ^1^H NMR spectrum of Dabcyl showed a singlet signal at 3.07 ppm, corresponding to the protons of the dimethylamino group, and the phenyl protons at 6.84, 7.81 and 8.05 ppm (Figure 4a). No noticeable shifts were found for these protons upon incremental addition of Pd^2+^, but new signals appeared at 6.69, 7.33 and 7.67 ppm, along with two new singlets at 3.11 and 3.20 ppm (Figure 4b, Figures S3 and S4).

This behaviour is close to that obtained by Babić et al. in the preparation of dicyclopalladated azobenzene derivatives [37]. In the cyclopalladation of azobenzene, the ligand binds to palladium through a coordinative bond from the lone electron pair of a nitrogen atom in the azo group. Then, a Pd-C bond is formed between the metal and an *ortho* carbon of the phenyl ring, resulting in a five-membered palladacycle [38,39]. Therefore, the appearance of the new aromatic signals in the ^1^H NMR spectrum of **2** could be ascribed to a loss of the *ortho* proton from each phenyl ring of **2** upon dicyclopalladation. As a result, the signals corresponding to the *ortho* and *meta* protons are shifted compared to the ion-free signal. These shifts are clearly visible when the ^1^H NMR spectrum is recorded at higher temperatures, where the *meta* protons H3′ and H6 appear at 6.53 ppm, and the H5′ and H2 protons at 6.69 ppm (Figure 4c). In addition, there is an increase in the proportion of a new complex with the increase in temperature, exhibiting a 1:1 ratio in the ^1^H NMR spectrum at 60 °C.

**Figure 5:**
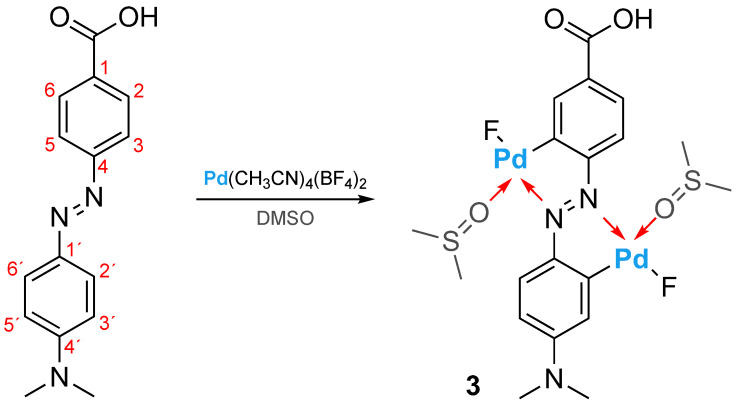
Suggested structure for complex **3** in DMSO solution.

In the case of the palladium tetrafluoroborate salt, fluoride abstraction from the tetrafluoroborate anion can also occur during cyclopalladation [40], suggesting the formation of a new complex, such as **3** (Figure 5), in DMSO-*d_6_* solution.

Furthermore, the shift observed in the methyl protons also indicates the coordination of palladium with the dimethylamino nitrogen. The protonation of this nitrogen atom decreases its donor strength, causing a downfield shift of the methyl signal. This result, along with the undefined isosbestic point observed in the UV-Vis titrations, suggests the presence of two protonation equilibria for chemosensor **2,** due to the coordination of palladium with the aniline nitrogen atom and the nitrogen atoms of the azo group.

On the other hand, it has been reported that azobenzene-based palladium (II) complexes can also form more complex structures in aqueous media [41,42]. For example, Luty-Błocho et al. demonstrated that methyl orange initially formed a palladacycle, which in time transformed into a H-shaped chloro-bridged dimer with a maximum in the UV-Vis spectrum at 559 nm and broad band between 600 and 800 nm (*λ*_max_ = 708 nm) in Britton-Robinson buffer solutions (pH = 4.1) [41].

**Figure 6:**
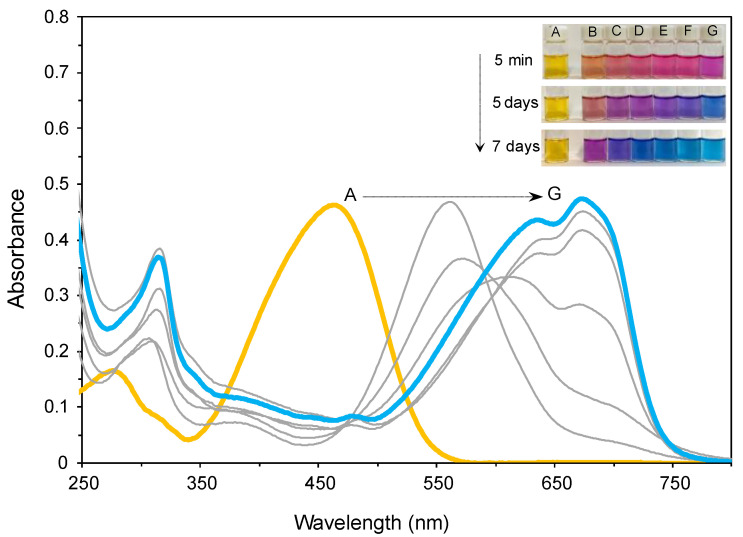
Absorption spectra of **2** in SDS (0.02 M, pH 6)–acetonitrile 99:1 (*v*/*v*) solution (2 × 10^−5^ M) after 7 days at different concentrations of Pd^2+^ (A: 0 equiv.; B: 0.5 equiv.; C: 1 equiv.; D: 2 equiv.; E: 3 equiv.; F: 4 equiv.; G: 5 equiv.).

**Figure 7:**
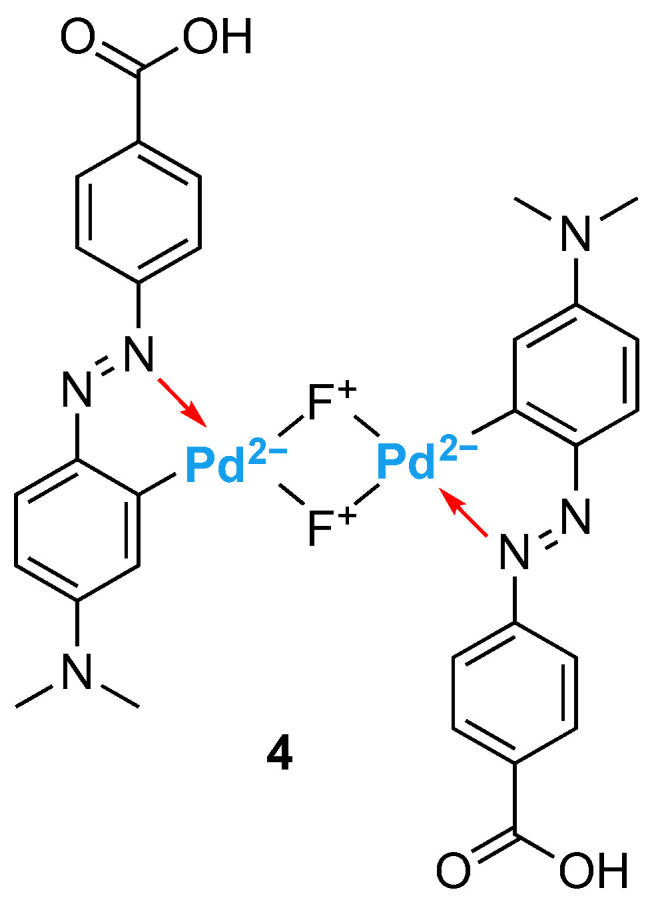
Suggested structure for complex **4**.

To better understand the complexation of **2** with Pd^2+^ in aqueous media, a similar study was conducted. Solutions of **2** (2 × 10^−5^ M) with different concentrations of Pd^2+^ (0–5 equivalents) were prepared, and the colour of the solutions, as well as the absorption spectra, were monitored over time. With time, there was a remarkable colour change in the solutions. Consequently, a new absorption band appeared at wavelengths 550–800 nm (*λ*_max_ = 674 nm) (Figure 6, the colours of the curves relate to the colours of the solutions). These results are close to those described in the literature [40,41,42]; thus, a difluoride-bridged palladium complex, **4**, was proposed for the interaction of **2** with Pd^2+^ in aqueous media (Figure 7). Also, the changes in absorbance of a solution of Dabcyl **2** with five equivalents of Pd^2+^ were monitored at different wavelengths (462, 555 and 674 nm) over a period of 7 days, and there seemed to be a stabilization 48 h after the addition of palladium (Figure S4).

#### 2.3.3. The Influence of pH

**Figure 8:**
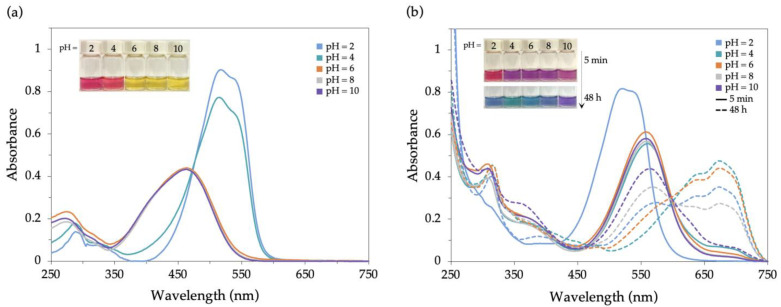
UV-Vis spectra and colour changes of **2** in SDS (0.02 M)–acetonitrile 99:1 (*v*/*v*) solution (2 × 10^−5^ M) in pH levels from 2 to 10 (**a**) before and (**b**) after 5 min (full line) and 48 h (broken line) upon the addition of 10 equiv. of Pd^2+^.

In order to investigate the effect of pH on the sensing properties of **2** for Pd^2+^, colorimetric studies were performed in SDS aqueous solutions (2 × 10^−5^ M) within the pH range of 2–10. In the absence of palladium, the Dabcyl solution showed a pink colour at pH 2 and 4 and a yellow colour at pH 6–10, confirming its pH-sensitive nature (Figure 8a). Remarkably, upon the addition of Pd^2+^ to these solutions, significant bathochromic shifts occurred in the ICT band of **2**, leading to noticeable colour changes visible to the naked eye (Figure 8b). As expected, this behaviour was enhanced over time, and although **2** was slightly more sensitive to Pd^2+^ at pH 4, there was no response to Sn^2+^ and Fe^3+^ at this pH level (see Section 2.4.3). These studies revealed that chemosensor **2** is very sensitive to palladium, even at different pH levels.

#### 2.3.4. Competition Assays

**Figure 9:**
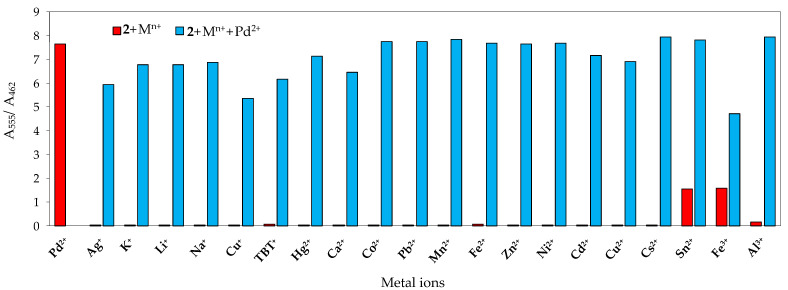
Absorbance ratio (A_555_/A_462_) of **2** (2 × 10^−5^ M) before and after the addition of 5 equiv. of Pd^2+^ in the presence of 10 equiv. background cations in SDS (0.02 M, pH 6)–acetonitrile 99:1 (*v*/*v*) solution.

Competition studies were conducted to evaluate the discrimination of chemosensor **2** to Pd^2+^ in the presence of other metal ions. As can be seen in Figure 9, the ratio between absorbance at 555 nm (λ_max_ for Pd^2+^-**2** complex) and at 462 nm (λ_max_ for free **2**) was plotted, and the spectral responses of **2** containing Pd^2+^ with the selected cations were similar to those of the sensor with only Pd^2+^, indicating that the presence of other ions does not interfere significantly with the complexation of **2** with Pd^2+^.

### 2.4. The Detection of Sn^2+^ and Fe^3+^ in SDS Aqueous Solution 

#### 2.4.1. UV-Vis Titrations

**Figure 10:**
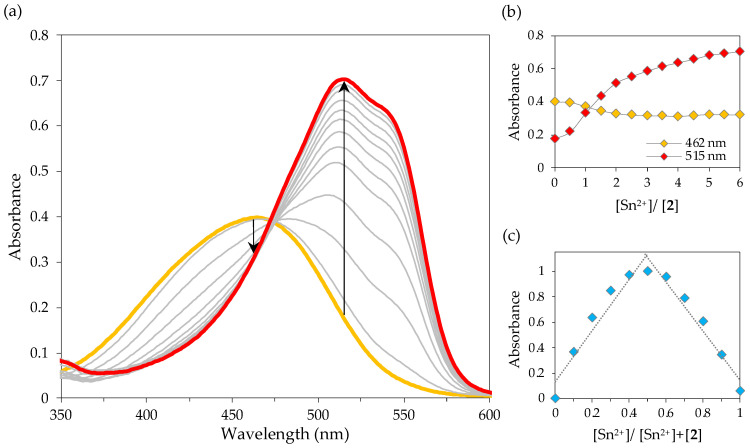
Absorption spectral changes of **2** with Sn^2+^ in SDS (0.02 M, pH 6)–acetonitrile 99:1 (*v*/*v*) solution (2 × 10^−5^ M): (**a**) UV-Vis titration of **2** upon gradual addition of Sn^2+^; (**b**) absorbance at 462 and 515 nm as a function of added Sn^2+^ equiv.; (**c**) Job’s plot for the complexation of **2** with Sn^2+^ at 515 nm.

**Figure 11:**
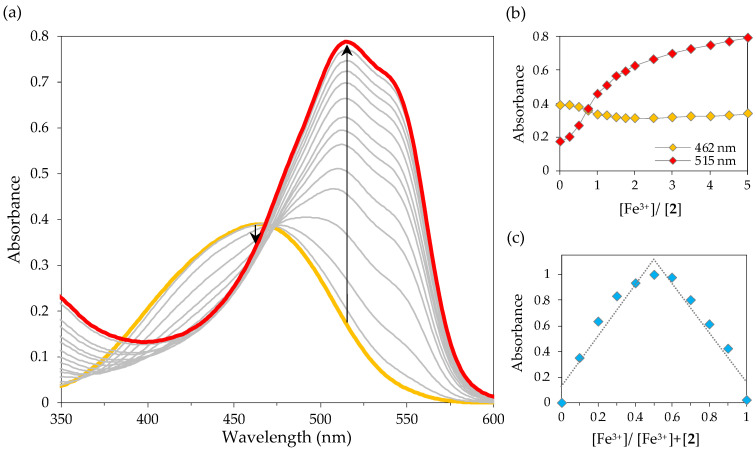
Absorption spectral changes of **2** with Fe^3+^ in SDS (0.02 M, pH 6)–acetonitrile 99:1 (*v*/*v*) solution (2 × 10^−5^ M): (**a**) UV-Vis titration of **2** upon gradual addition of Fe^3+^; (**b**) absorbance at 462 and 515 nm as a function of added Fe^3+^ equiv.; (**c**) Job’s plot for the complexation of **2** with Fe^3+^ at 515 nm.

The response of chemosensor **2** towards Sn^2+^ and Fe^3+^ was also evaluated by UV-Vis titrations in SDS (0.02 M, pH 7.5)–acetonitrile 99:1 (*v*/*v*) solution. Notably, the gradual addition of Sn^2+^ and Fe^3+^ ions induced a similar response in the UV-Vis spectrum of chemosensor **2**: the absorption band at 462 nm slightly decreased, while a new peak at 515 nm increased significantly, with an isosbestic point at 472 nm (Figure 10a and Figure 11a, the colours of the curves relate to the colours of the solutions).

The addition of five equivalents of Fe^3+^ resulted in a higher increase of the absorbance at 515 nm compared to that observed upon interaction with six equivalents of Sn^2+^, indicating that chemosensor **2** is more sensitive to iron (Figure 10b and Figure 11b). In fact, the detection limit for Sn^2+^ was found to be 1.3 × 10^−7^ M, while the DL for Fe^3+^ was estimated to be 5.2 × 10^−8^ M. These values are below the WHO limits for tin and iron in drinking water (8 × 10^−4^ M to 8 × 10^−3^ M and 5.4 × 10^−6^ M, respectively) [11,43] and the maximum acceptable level of tin (2.1 × 10^−6^ M) in canned food [44]. In addition, the detection limits obtained for Sn^2+^ and Fe^3+^ are quite comparable to the sensors described in the literature for these metal ions (Table 2).

Job’s plot analysis (Figure 10c and Figure 11c) confirmed the formation of complexes with ligand to metal 1:1 stoichiometry (**2**:Sn^2+^ and **2**:Fe^3+^).

#### 2.4.2. ^1^H NMR Studies

**Figure 12:**
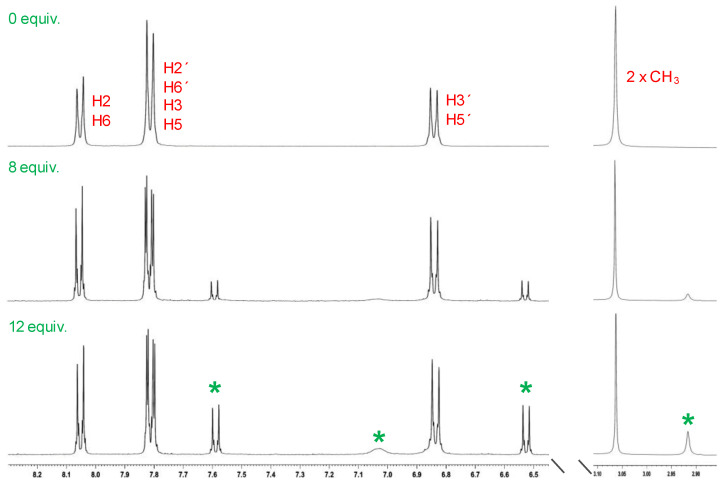
Partial ^1^H NMR spectra of **2** with Sn^2+^ in DMSO-*d_6_* at 25 °C (* indicates the new signals).

To better understand the complexation of **2** with Sn^2+^, ^1^H NMR titrations were conducted in a DMSO-*d_6_* solution. As mentioned above, in the ^1^H NMR spectrum of Dabcyl **2**, the signal of the methyl groups was observed at 3.07 ppm, and the aromatic signals at 6.84, 7.81 and 8.05 ppm. Upon incremental addition of Sn^2+^, new peaks appeared at 2.91, 6.52, 7.02 and 7.59 ppm (Figure 12). These results suggest that each nitrogen atom of the azo moiety could be coordinating with the tin atom through a mechanism similar to that described for the complexation of Dabcyl with Pd^2+^. The appearance of new signals in the NMR titration with Sn^2+^ at lower chemical shifts than those observed in the NMR titration with Pd^2+^ (6.60, 7.33 and 7.67 ppm) strongly indicate that tin complexation only takes place at the azo moiety and does not affect the aniline nitrogen. Due to the paramagnetic nature of Fe^3+^, ^1^H NMR titrations were not performed for this metal ion.

#### 2.4.3. The Influence of pH

The effect of pH on the sensing ability of **2** towards Sn^2+^ and Fe^3+^ was also investigated in SDS aqueous solutions in the pH range of 2–10. Upon the addition of these cations, the absorbance of chemosensor **2** remained almost unchanged at pH < 4 and pH = 10 (Figures S5 and S6). However, we observed a slightly bathochromic shift in the UV-Vis spectrum of **2** at pH 8. As expected, the absorbance at 515 nm significantly increased at pH 6, accompanied by a remarkable colour change from yellow to magenta. These results confirmed that chemosensor **2** exhibits sensitivity for Sn^2+^ and Fe^3+^ at pH 6. Overall, when comparing to the results for Pd^2+^ (see Figure 8), the most naked-eye-perceptible change occurs for Pd^2+^, from yellow to greenish-blue.

#### 2.4.4. Competition Assays

**Figure 13:**
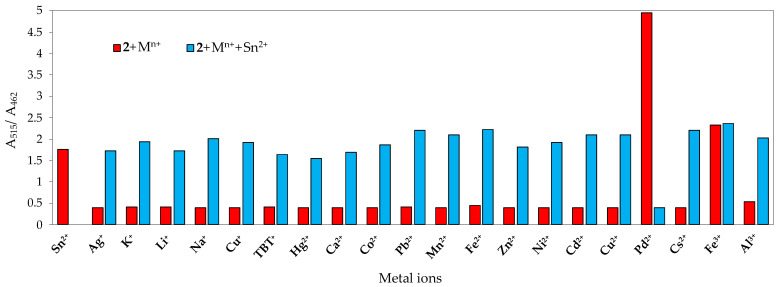
Absorbance ratio (A_515_/A_462_) of **2** (2 × 10^−5^ M) before and after the addition of 5 equiv. of Sn^2+^ in the presence of 10 equiv. background metal ions in SDS (0.02 M, pH 6)–acetonitrile 99:1 (*v*/*v*) solution.

**Figure 14:**
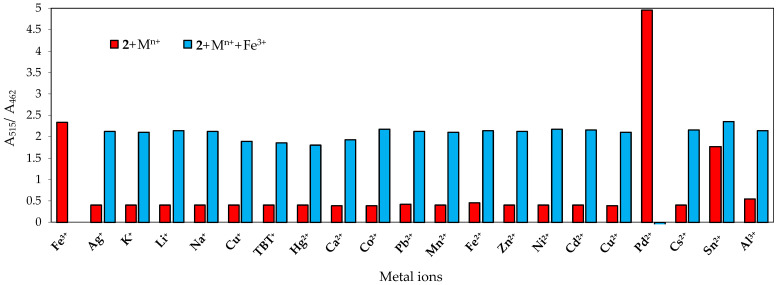
Absorbance ratio (A_515_/A_462_) of **2** (2 × 10^−5^ M) before and after the addition of 5 equiv. of Fe^3+^ in the presence of 10 equiv. background metal ions in SDS (0.02 M, pH 6)–acetonitrile 99:1 (*v*/*v*) solution.

To further confirm the interaction of chemosensor **2** with Sn^2+^ and Fe^3+^, competition experiments were performed by recording the absorption spectra of chemosensor **2** in the presence of five equivalents of Sn^2+^ and Fe^3+^ ions with 10 equivalents of the other metal ions. The ratio between absorbance at 515 nm (λ_max_ for Sn^2+^-**2** complex and Fe^3+^-**2** complex) and at 462 nm (λ_max_ for free **2**) was plotted, and, not surprisingly, a selective colorimetric behaviour was seen for Pd^2+^ (Figure 13 and Figure 14). The other ions exhibited a similar absorbance ratio A_515_/A_462_ to those of **2** in the presence of Sn^2+^ and Fe^3+^. Therefore, it was confirmed that chemosensor **2** had high selectivity towards Pd^2+^ over other metal ions in SDS aqueous mixtures.

## 3. Materials and Methods

### 3.1. Materials and Instruments

All the chemicals and solvents were purchased from Acros Organics (Geel, Belgium) and Sigma-Aldrich (St. Louis, MO, USA) and were used as received. The synthesis and structural characterization of Dabcyl acid **2** has been reported by us elsewhere [27]. UV-Vis absorption spectra were collected using a Shimadzu UV/2501PC spectrophotometer (Shimadzu Europa GmbH, Duisburg, Germany) in standard quartz cuvettes with a 1 cm optical path. NMR spectra were recorded on a Bruker Avance III 400 at an operating frequency of 400 MHz for ^1^H and 100.6 MHz for ^13^C, using the solvent peak as the internal reference at 25 °C; the chemical shift values (*δ* relative to TMS) are given in ppm.

### 3.2. Stock Solutions

Stock solutions (1 × 10^−2^ M) of the hydrated tetrafluorborate salts (Li^+^, Cu^+^, Co^2+^ and Pd^2+)^, chloride salts (TBT^+^ and Sn^2+^) and hydrated perchlorate salts (Ag^+^, K^+^, Na^+^, Hg^2+^, Ca^2+^, Pb^2+^, Mn^2+^, Fe^2+^, Zn^2+^, Ni^2+^, Cd^2+^, Cu^2+^, Cs^2+^, Fe^3+^ and Al^3+^) were prepared in UV-grade acetonitrile. A solution of compound **2** (2 × 10^−5^ M) was prepared in SDS (0.02 M, pH 6)–acetonitrile 99:1 (*v*/*v*).

### 3.3. Preliminary Chemosensing Tests and UV-Vis Titrations

Preliminary chemosensing studies were performed by the addition of 10 equivalents of each cation to the solution of compound **2**, and the colorimetric responses were evaluated by the naked eye and by recording the UV-Vis spectra of these solutions. UV-Vis titrations were carried out by the gradual addition of each cation to the solution of **2** (3 mL), and the absorption spectra were recorded until reaching the maximum optical change. Scans were collected after 3 min of each cation addition.

### 3.4. Determination of the Detection Limit (DL)

The absorption spectrum of **2** was measured five times to obtain the standard division of the absorbance. Then, the detection limit (DL) was calculated using the equation DL = 3*σ*/*S*, where *σ* is the standard division of the blank solution measurement mentioned above and *S* is the slope of the linear plot of the concentration-dependent absorbance response [30]. 

### 3.5. Binding Studies

Job’s plots were determined by recording the absorbance of various mole fractions of **2** and the corresponding cation at 515 nm (for Sn^2+^ and Fe^3+^) and 555 nm (for Pd^2+^). The signal was plotted as a function of the molar ratio [M^n+^]/[M^n+^] + [2]. The total concentration of **2** and the cations under study was 2 × 10^—5^ M. NMR titrations were carried out by gradual addition of each cation (6 × 10^−1^ M) to the solution of **2** (1 × 10^−2^ M) in DMSO-*d_6_* at 25, 40 and 60 °C.

## 4. Conclusions

In summary, we report that an azo dye-based chemosensor **2** for metal ions showed various advantageous properties as a colorimetric chemosensor, such as a high molar absorption coefficient (*ε*_max_ = 19,000 M^—1^ cm^—1^) at 462 nm in SDS aqueous mixtures at pH 6; a remarkable colour change visible to the naked eye, from yellow to purple (after 5 min) or greenish-blue (after 48 h) for Pd^2+^ and from yellow to magenta in the case of Sn^2+^ and Fe^3+^); high sensitivity for Pd^2+^, Sn^2+^ and Fe^3+^, with low detection limits (5.4 × 10^−8^ M, 1.3 × 10^−7^ M and 5.2 × 10^−8^ M, respectively); and the ability to monitor such ions even in the presence of other cations. Therefore, compound **2** could be used as an efficient colorimetric chemosensor for the detection of Pd^2+^, Sn^2+^ and Fe^3+^ in industrial and environmental samples containing other metal ions in aqueous media. 

## Figures and Tables

**Table 1:** Comparison of **2** with reported chemosensors for Pd^2+^ detection.

Reported Sensors	DL (M)	Method	Reference
Azo dye **2**	5.4 × 10^−8^	UV-Vis	This work
Rhodamine-based chemosensor	2.8 × 10^−7^	UV-Vis	[32]
Naphthaldehyde functionalized Schiff base	9.8 × 10^−7^	UV-Vis	[33]
Sulphur-containing rhodamine-based probe	6.3 × 10^−9^	UV-Vis	[34]
Azo dye-rhodamine-based chemosensor	4.5 × 10^−7^	Fluorescence	[35]
Coumarin-based probe	4.1 × 10^−8^	Fluorescence	[36]

**Table 2:** Comparison of **2** with reported chemosensors for Sn^2+^ and Fe^3+^ detection.

Reported Probes	DL (M)	Method	Reference
Sn^2+^			
Azo dye **2**	1.3 × 10^−7^	UV-Vis	This work
Azo-linked Schiff base	7.5 × 10^−7^	UV-Vis	[45]
Rhodamine-triazole-pyridine chemosensor	1.2 × 10^−7^	UV-Vis	[46]
Rhodamine-based chemosensor	5.1 × 10^−6^	UV-Vis	[47]
Naphthoquinone-based chemosensor	1.1 × 10^−7^	Fluorescence	[48]
Naphthalene-based probe	2.6 × 10^−8^	Fluorescence	[49]
Fe^3+^			
Azo dye **2**	5.2 × 10^−8^	UV-Vis	This work
Azo dye–rhodamine-based chemosensor	5.1 × 10^−6^	UV-Vis	[50]
Rhodamine-based chemosensor	4.0 × 10^−8^	UV-Vis	[51]
Naphthalenediimide-based probe	1.0 × 10^−7^	UV-Vis	[52]
Sensor-based azo compound	1.8 × 10^−8^	UV-Vis	[53]
Coumarin Schiff base derivatives	4.8 × 10^−7^	Fluorescence	[54]

## Data Availability

Not applicable.

## Supplementary Materials

The following supporting information can be downloaded at: https://www.mdpi.com/article/10.3390/molecules28166111/s1, Figure S1: ^1^H NMR spectrum of compound **2** (DMSO-*d_6_*, 400 MHz, 25 °C). Figure S2: ^13^C NMR spectrum of compound **2** (DMSO-*d_6_*, 100.6 MHz, 25 °C). Figure S3: Partial ^1^H NMR spectra of compound **2** with increasing number of equivalents of Pd^2+^ (DMSO-*d_6_*, 400 MHz, 25 °C). Figure S4: Changes in absorbance at diferent wavelengths (462, 555 and 674 nm) over time for the interaction of Dabcyl **2** (2 × 10^−5^ M) with Pd^2+^ (5 equiv.) in SDS (0.02 M, pH 6)-acetonitrile 99:1 (*v*/*v*). Figure S5: Spectral (a) and colorimetric (b) changes of **2** with Sn^2+^ (10 equiv.) in SDS aqueous solution (2 × 10^−5^ M) with a variation of pH from 2 to 10. Figure S6: Spectral (a) and colorimetric (b) changes of **2** with Fe^3+^ (10 equiv.) in SDS aqueous solution (2 × 10^−5^ M) with a variation of pH from 2 to 10.
