# Supplementary material for: Dabcyl as a Naked Eye Colorimetric Chemosensor for Palladium Detection in Aqueous Medium
Source: Molecules. 2023 Aug 17;28(16):6111. doi: 10.3390/molecules28166111 (PMC10459738; doi:10.3390/molecules28166111)
Supplement: Supplementary file 1 [file molecules-28-06111-s001.zip › molecules-2519063-supplementary.pdf]

Dabcyl as a Naked Eye Colorimetric Chemosensor for Palladium Detection in Aqueous Medium

Cátia D. F. Martins, M. Manuela M. Raposo, Susana P. G. Costa

Centre of Chemistry, University of Minho, Campus de Gualtar, 4710-057 Braga, Portugal

Supplementary Material

Table of Contents:

Figure S1. ^{1}H NMR spectrum of compound **2** (DMSO-d_6, 400 MHz, 25 °C).

Figure S2. ^{13}C NMR spectrum of compound **2** (DMSO-d_6, 100.6 MHz, 25 °C).

Figure S3. Partial ^{1}H NMR spectra of compound **2** with increasing number of equivalents of Pd^{2+} (DMSO-d_6, 400 MHz, 25 °C).

Figure S4. Changes in absorbance at diferent wavelengths (462, 555 and 674 nm) over time for the interaction of Dabcyl **2** (2×10^{-5} M) with Pd^{2+} (5 equiv.) in SDS (0.02 M, pH 6)-acetonitrile 99:1 (v/v).

Figure S5. Spectral (a) and colorimetric (b) changes of **2** with Sn^{2+} (10 equiv.) in SDS aqueous solution (2×10^{-5} M) with a variation of pH from 2 to 10.

Figure S6. Spectral (a) and colorimetric (b) changes of **2** with Fe^{3+} (10 equiv.) in SDS aqueous solution (2×10^{-5} M) with a variation of pH from 2 to 10.

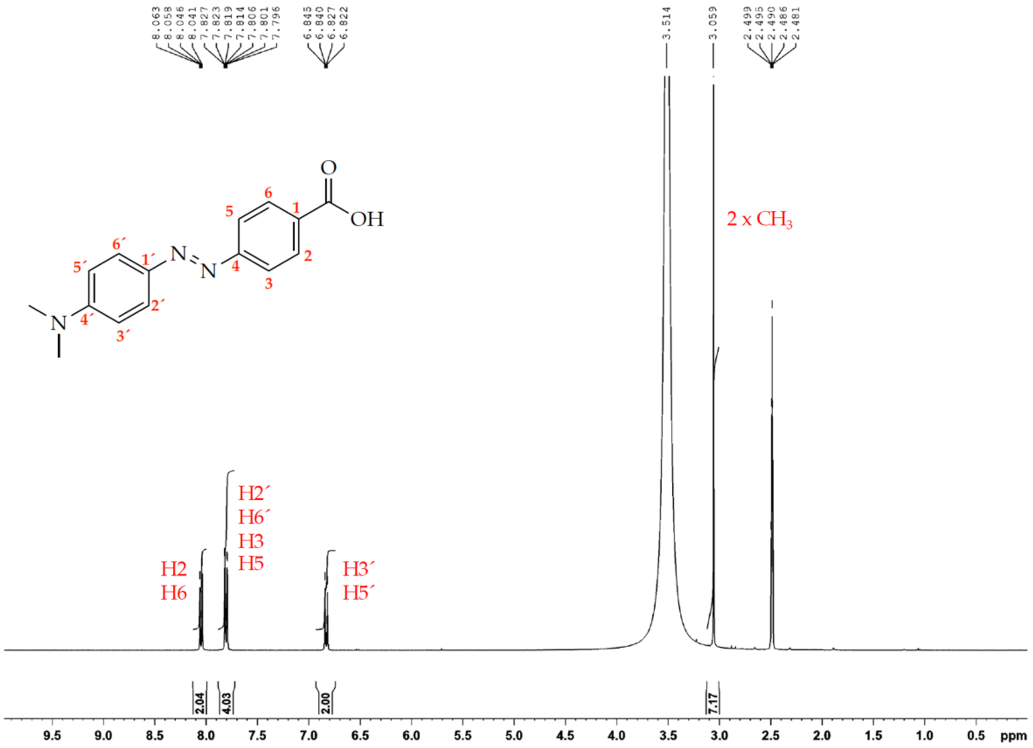

Figure S1. ^{1}H NMR spectrum of compound **2** (DMSO-d_6, 400 MHz, 25 °C).

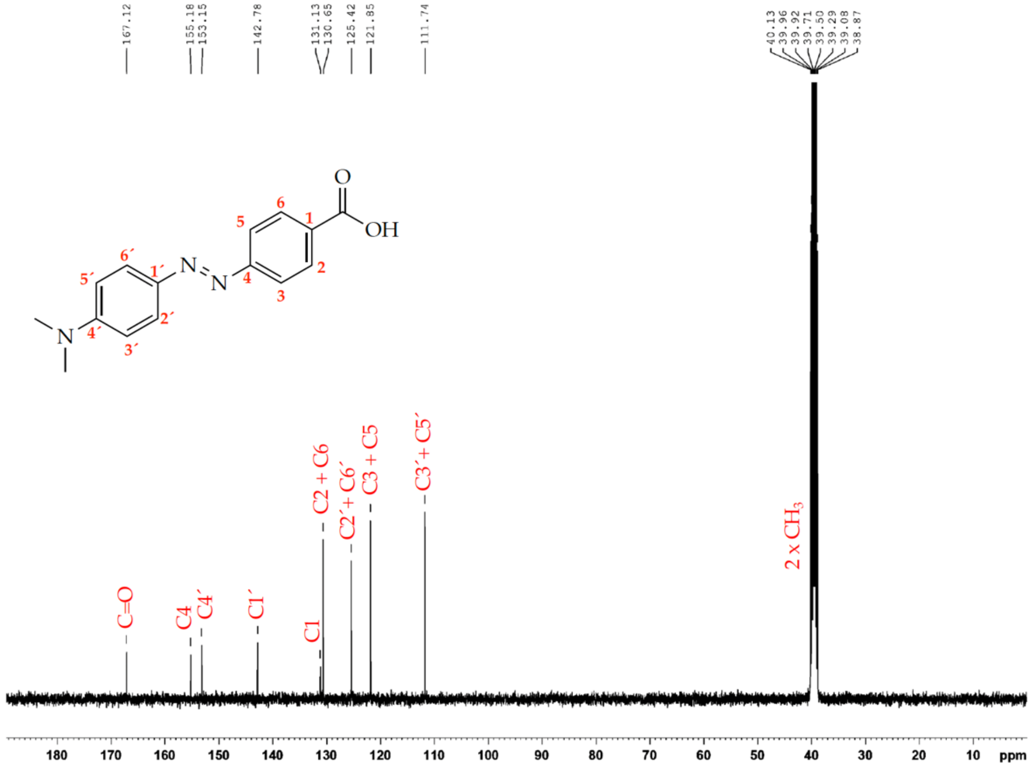

Figure S2. ^{13}C NMR spectrum of compound **2** (DMSO-d_6, 100.6 MHz, 25 °C).

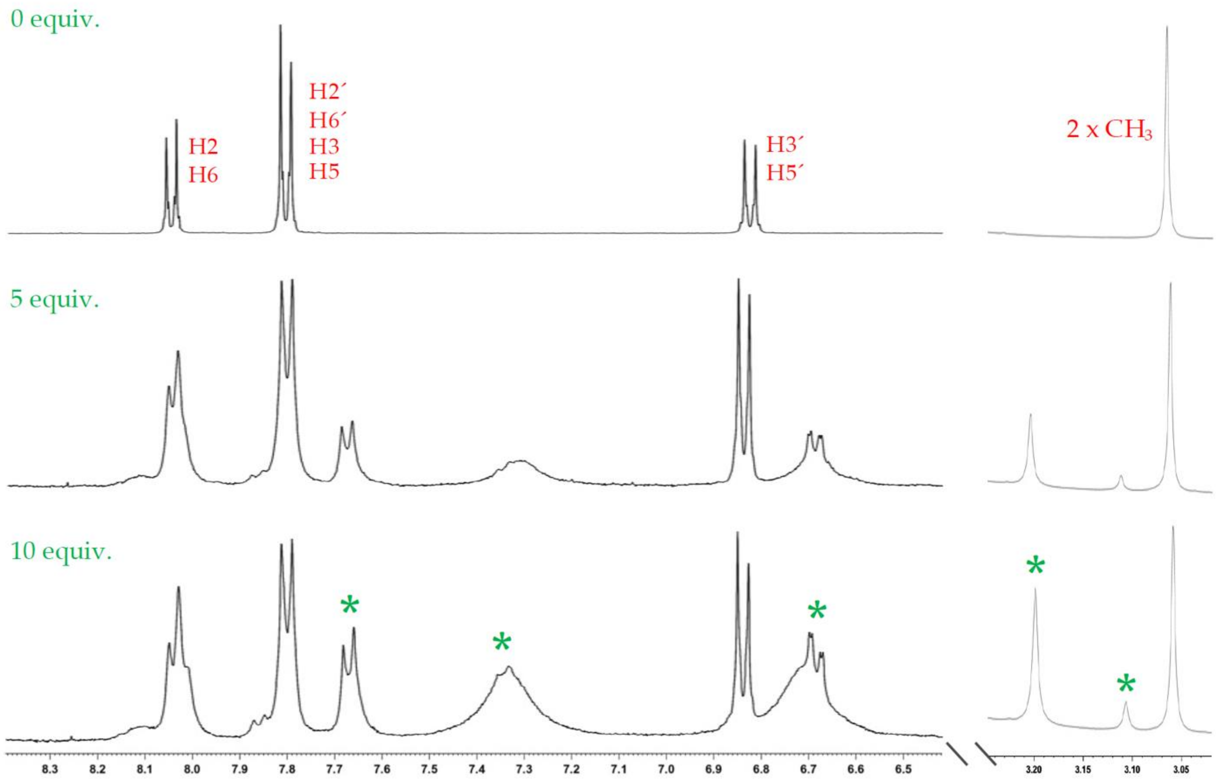

Figure S3. Partial ^{1}H NMR spectra of compound **2** with increasing number of equivalents of Pd^{2+} in DMSO-d_6 at 25 ºC (* indicates the new signals).

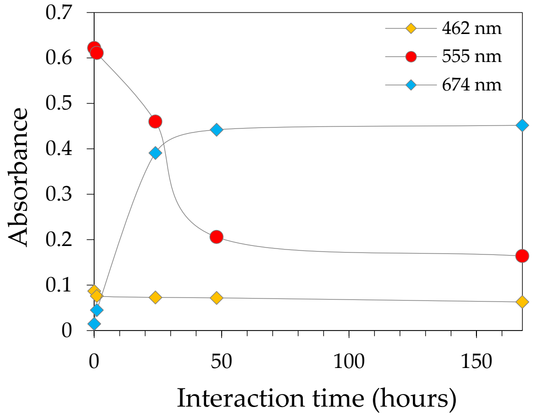

Figure S4. Changes in absorbance at diferent wavelengths (462, 555 and 674 nm) over time for the interaction of Dabcyl **2** (2×10^{-5} M) with Pd^{2+} (5 equiv.) in SDS (0.02 M, pH 6)-acetonitrile 99:1 (v/v).

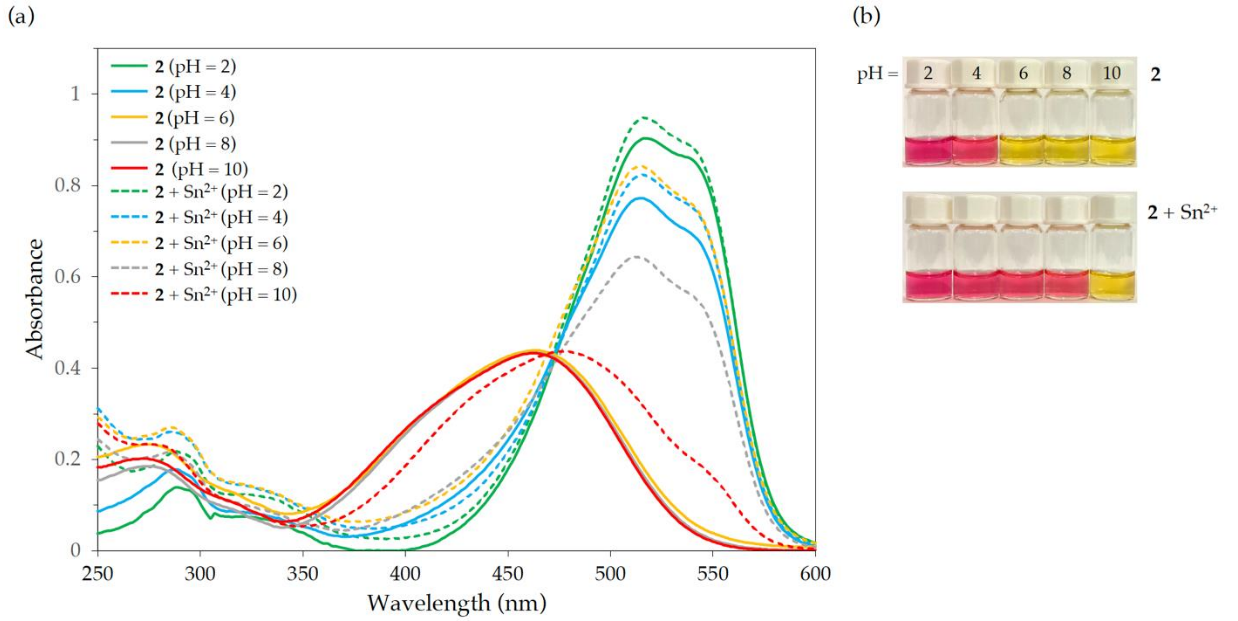

Figure S5. Spectral (a) and colorimetric (b) changes of **2** with Sn^{2+} (10 equiv.) in SDS aqueous solution (2 × 10^{-5} M) with a variation of pH from 2 to 10.

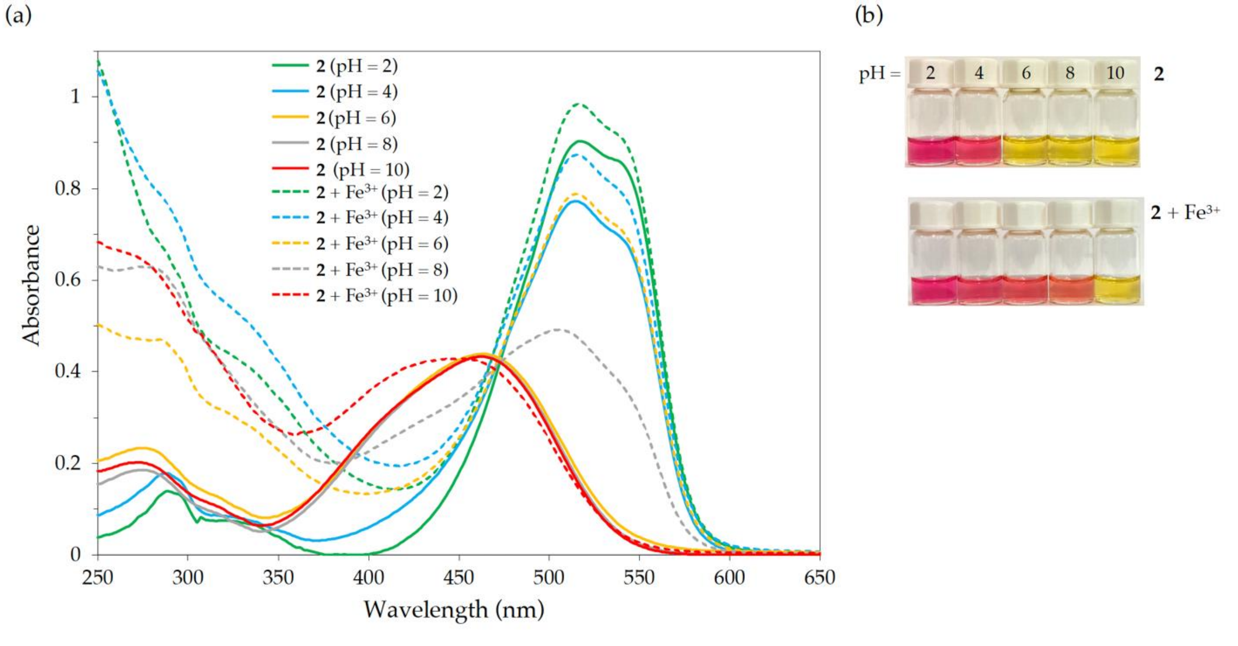

Figure S6. Spectral (a) and colorimetric (b) changes of **2** with Fe^{3+} (10 equiv.) in SDS aqueous solution (2 × 10^{-5} M) with a variation of pH from 2 to 10.
